# Broad and Protective Influenza B Virus Neuraminidase Antibodies in Humans after Vaccination and their Clonal Persistence as Plasma Cells

**DOI:** 10.1128/mBio.00066-19

**Published:** 2019-03-12

**Authors:** Michael S. Piepenbrink, Aitor Nogales, Madhubanti Basu, Christopher F. Fucile, Jane L. Liesveld, Michael C. Keefer, Alexander F. Rosenberg, Luis Martinez-Sobrido, James J. Kobie

**Affiliations:** a Infectious Diseases Division, University of Rochester, Rochester, New York, USA; b Department of Microbiology & Immunology, University of Rochester, Rochester, New York, USA; c Informatics Institute, University of Alabama at Birmingham, Birmingham, Alabama, USA; d Division of Hematology/Oncology/James P. Wilmot Cancer Institute, University of Rochester, Rochester, New York, USA; e Department of Microbiology, University of Alabama at Birmingham, Birmingham, Alabama, USA; Columbia University Medical College

**Keywords:** B cell responses, human, influenza vaccines, monoclonal antibodies, neuraminidase

## Abstract

Although most seasonal inactivated influenza vaccines (IIV) contain neuraminidase (NA), the extent and mechanisms of action of protective human NA-specific humoral responses induced by vaccination are poorly resolved. Due to the propensity of influenza virus for antigenic drift and shift and its tendency to elicit predominantly strain-specific antibodies, humanity remains susceptible to waves of new strains of seasonal viruses and is at risk from viruses with pandemic potential for which limited or no immunity may exist. Here we demonstrate that the use of IIV results in increased levels of influenza B virus (IBV) NA-specific serum antibodies. Detailed analysis of the IBV NA B cell response indicates concurrent expansion of IBV NA-specific peripheral blood plasmablasts 7 days after IIV immunization which express monoclonal antibodies with broad and potent antiviral activity against both IBV Victoria and Yamagata lineages and prophylactic and therapeutic activity in mice. These IBV NA-specific B cell clonal lineages persisted in CD138^+^ long-lived bone marrow plasma cells. These results represent the first demonstration that IIV-induced NA human antibodies can protect and treat influenza virus infection *in vivo* and suggest that IIV can induce a subset of IBV NA-specific B cells with broad protective potential, a feature that warrants further study for universal influenza vaccine development.

## Abstract (precis)

Influenza virus infections continue to cause substantial morbidity and mortality despite the availability of seasonal vaccines. The extensive genetic variability in seasonal and potentially pandemic influenza strains necessitates new vaccine strategies that can induce universal protection by focusing the immune response on generating protective antibodies against conserved targets such as regions within the influenza neuraminidase protein. We have demonstrated that seasonal immunization stimulates neuraminidase-specific antibodies in humans that are broad and potent in their protection from influenza B virus when tested in mice. These antibodies further persist in the bone marrow, where they are expressed by long-lived antibody-producing cells, referred to here as plasma cells. The significance in our research is the demonstration that seasonal influenza immunization can induce a subset of neuraminidase-specific B cells with broad protective potential, a process that if further studied and enhanced could aid in the development of a universal influenza vaccine.

## INTRODUCTION

Although vaccination is still the best protection against influenza, circulating virus strains are not always predictable in a given year. Current quadrivalent vaccines include influenza A virus (IAV) H1N1 and H3N2 and influenza B virus (IBV) from both the Victoria and Yamagata lineages. The incidence and severity of IBV infection vary; however, children are often particularly highly impacted (1–5). The level of influenza-associated pediatric deaths attributed to IBV in the United States was quite high in the 2012 to 2013 season at 52% of the fatal pediatric influenza cases, while the average level during the last 10 years was 27% (6, 7). Furthermore, the neuraminidase (NA) inhibitor oseltamivir has also been shown to be less effective in children infected with IBV, and zanamivir, another NA inhibitor, is not approved for use in children less than 7 years of age, highlighting the particular vulnerability of children to IBV infections (6, 8). Public health concerns posed by IBV are further heightened by the lack of activity of M2 inhibitors (e.g., amantadine and rimantadine) (9–11). A new drug, baloxavir marboxil (Xofluza), has been recently approved by the Food and Drug Administration (FDA) for the treatment of acute uncomplicated influenza in patients who have been symptomatic for no more than 48 h (12).

The immune response to influenza virus is dominated by the production of hemagglutinin (HA) antibodies (Abs). The yearly mutation rate among influenza HA proteins is 1% to 2%, leading to development of new strains of influenza virus that are resistant to previous immunity. The other most predominant glycoprotein on the virion surface is NA. Although immunogenic, the predominance of NA-specific antibodies is much lower than that of HA antibodies, probably because NA represents only one-fourth the amount of the HA on the virion surface (13). The yearly rate of mutation for NA is lower than for HA (14–16), while part of the enzymatic site (ILRTQESEC) remains conserved across IAV and IBV (17), making NA a potentially effective target for universal vaccine and therapeutic human monoclonal Ab (hMAb) development.

NA catalyzes the cleavage of terminal sialic acids from a large variety of glycoproteins, glycolipids, and oligosaccharides (18, 19), with human isolates primarily exhibiting more-efficient cleavage of α2-3 linked sialic acid than α2-6 sialic acid (20, 21). NA is important during the final stages of influenza virus infection, where it removes sialic acid from infected cell surfaces and newly formed virions, thus facilitating progeny virus release and spread of the infection to neighboring cells (22, 23). NA can also promote penetration of the virus through the ciliated epithelium of the human airway by removing sialic acids on mucins, cilia, and the cellular glycocalyx (24). Thus, unlike antibodies against HA, antibodies against NA do not seem to be directly neutralizing influenza but rather prevent the spread of influenza viruses from infected cells by blocking the activity of the enzymatic site. Additionally, NA-specific Abs may also help clear virus through the engagement of the Fc region, thus mediating complement activation, antibody-dependent cellular cytotoxicity (ADCC), and antigen-dependent cellular phagocytosis (ADCP) (25–31).

The correlation of NA-specific Ab with protection from influenza virus infection in humans has been demonstrated by multiple studies, including those investigating H1N1 (32, 33) and H3N2 (34–36) viruses. Higher titers of serum NA-inhibiting antibodies were found to be associated with decreased levels of infection incidence, symptom severity, and viral shedding in an H1N1 human challenge study (33). Further, numerous animal studies have directly demonstrated that NA-specific Abs can prevent and treat influenza virus infection, using either active immunization with NA or passive immunization with NA-specific polyclonal or monoclonal Abs (37–42). The precise features of the human NA-specific B cell response to IIV, particularly the potential of IIV-induced NA-specific antibodies to directly mediate protection from influenza virus infection, remain poorly resolved.

Here we demonstrated that the seasonal IIV induces IBV NA-specific serum antibodies and B cells and isolated hMAbs that exhibit broad and potent *in vitro* and *in vivo* viral inhibition against IBV. Our results also demonstrate the feasibility of targeting IBV NA with hMAbs for the therapeutic treatment of IBV infections.

## RESULTS

### Seasonal influenza vaccine induces IBV NA-specific antibody and plasmablasts.

**FIG 1:**
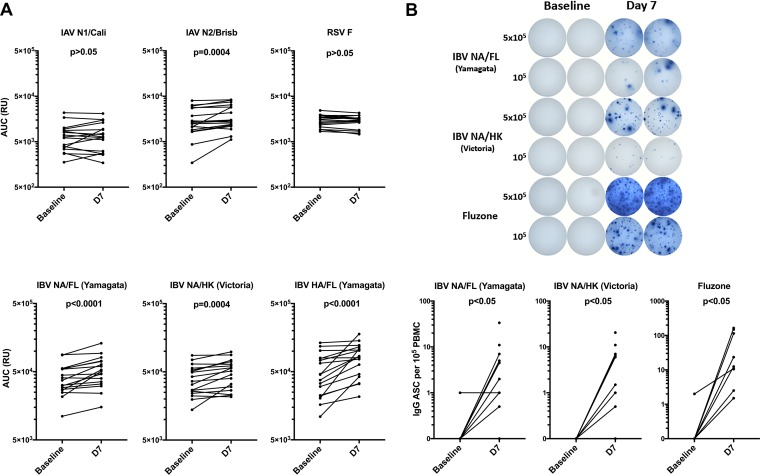
Increased levels of IBV NA-specific plasma antibodies and plasmablasts after IIV immunization. Peripheral blood was collected at baseline and at day 7 (D7) after immunization with IIV. (A) Plasma was serially diluted, IgG specific for NA, HA, and RSV F proteins was detected by ELISA, and area under the curve (AUC) data (*n* = 17 subjects) are presented. (B) IBV NA and IIV (Fluzone) IgG-specific ELISpot assays were performed on total PBMC (*n* = 11 subjects). Representative ELISpot assay from a single subject displayed (top). Symbols represent individual subjects. Significance determined by paired *t* test.

Peripheral blood samples were obtained from healthy adult subjects prior to (baseline) and 7 days after (D7) receiving the 2014-to-2015 seasonal quadrivalent IIV. Overall, significant increases (*P* < 0.05) in levels of IAV N2 A/Wisconsin/67/2005-specific, IBV NA B/Hong Kong/330/2001 (Victoria lineage)-specific, and IBV HA B/Florida/04/2006 (Yamagata lineage)-specific virus plasma IgG binding antibodies were observed, primarily driven by a subset of subjects whose titers increased following immunization; however, the titers in isolates from many subjects did not increase. IAV N1 A/California/04/2009-specific plasma IgG levels increased in 41% of subjects, but the results did not reach overall statistical significance (Fig. 1A). As expected, no significant increase in the levels of respiratory syncytial virus (RSV) fusion (F) protein-specific plasma IgG was observed following IIV immunization. Further evaluation of the IBV NA-specific response revealed a significant (*P* < 0.05) expansion of peripheral blood plasmablasts secreting IgG specific for IBV NA B/Hong Kong/330/2001 (Victoria lineage) and NA B/Florida/04/2006 (Yamagata lineage) viruses at D7, although the level was substantially lower than that of the overall IIV-specific plasmablast response (Fig. 1B). These results demonstrate that IIV can induce an IBV NA-specific humoral response in humans.

### IBV NA-specific plasmablasts include high-affinity broadly reactive monoclonal antibodies.

**FIG 2:**
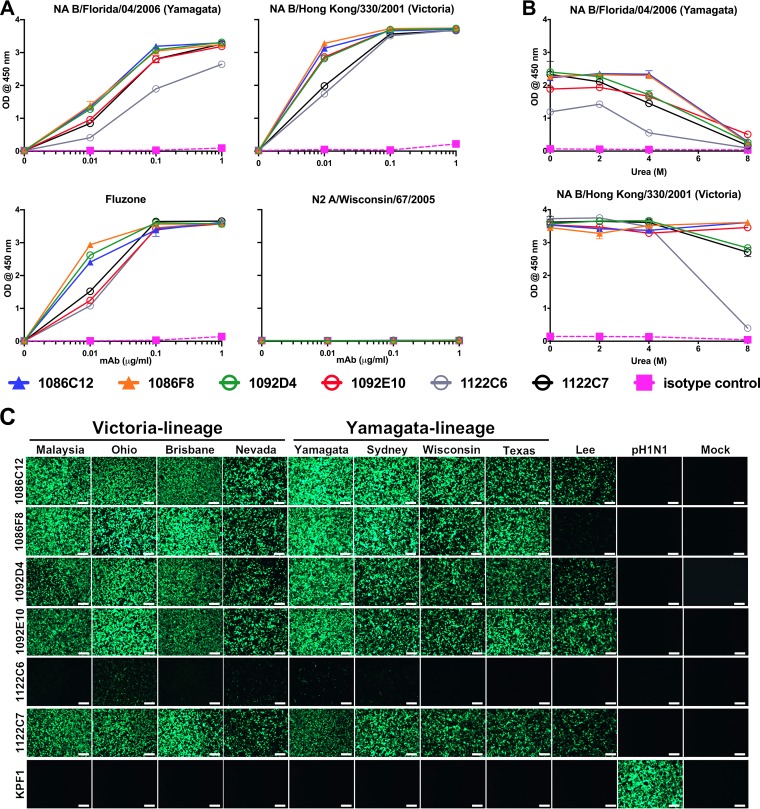
IBV NA-specific hMAbs recognize Victoria and Yamagata lineages. IBV NA-specific hMAbs were generated from plasmablasts following IIV immunization. (A) Increasing concentrations of hMAbs were tested for binding to the indicated NA proteins and IIV (Fluzone) by ELISA. RU, relative units. (B) hMAbs were tested for avidity for NA proteins at 1 μg/ml in increasing concentrations of urea. (C) MDCK cells were mock infected (Mock) or infected (MOI of 0.1) with the indicated viruses and 17 h later were fixed and stained with 1 μg/ml of the NA-specific hMAbs and NA protein expression evaluated by IFA. KPF1 is an H1-specific hMAb used as an internal control in this IFA. Bar, 100 µm. Designations used in the figure are as follows: Malaysia, B/Malaysia/2506/2004; Ohio, B/Ohio/01/2005; Brisbane, B/Brisbane/60/2008; Nevada, B/Nevada/03/2011; Yamagata, B/Yamagata/16/1988; Sydney, B/Sydney/507/2006; Wisconsin, B/Wisconsin/01/2010; Texas, B/Texas/06/2011; Lee, B/Lee/1940; pH1N1, A/California/4_NYICE_E3/2009.

To define the characteristics and functional potential of IIV-induced IBV NA-specific antibodies, D7 plasmablasts were sorted as single cells from two subjects (105 and 134) who demonstrated increased levels IBV NA-specific plasma IgG, and their expressed immunoglobulin heavy chain and light chain variable regions were cloned to generate recombinant fully hMAbs. Subsequently, six IBV NA-specific hMAbs were isolated that exhibited strong binding to recombinant IBV NA protein from the Victoria (B/Hong Kong/330/2001) and Yamagata (B/Florida/04/2006) virus lineages, in addition to the 2016/2017 IIV (Fluzone) (Fig. 2A). No reactivity to IAV N2 was detected, indicating the highly specific binding of these IBV NA hMAbs. The stability of the binding of the hMAbs to NA was accessed by treatment with increasing urea concentrations. All hMAbs maintained greater than 75% of their binding activity against IBV NA B/Hong Kong/330/2001 in 8 M urea treatment, with the exception of 1122C6, which had reduced binding stability. These hMAbs similarly maintained their binding activity against NA B/Florida/04/2006 in 4 M urea, but the level was substantially diminished in 8 M urea (Fig. 2B). Binding affinity was further tested using the 1092D4 hMAb and surface plasmon resonance (see Fig. S1 in the supplemental material). 1092D4 bound both B/Hong Kong/330/2001 and B/Florida/04/2006 NA proteins with very high affinity; specifically, the equilibrium dissociation constants (*K_d_*) of binding were 100 and 185 pM, respectively.

10.1128/mBio.00066-19.1 FIG S1 Surface plasmon resonance of MAb 1092D4. Purified 1092D4 was captured on a protein G chip with NA from either B/Florida/04/2006 (top panel) or B/Hong Kong/330/2001 (bottom panel) at decreasing concentrations with passage over each channel. The data points are shown in black, and the data corresponding to the fit to a 1:1 binding model are shown in red. The results of one experiment representative of two (B/Florida/04/2006) or three (B/Hong Kong/330/2001) are presented. Download FIG S1, TIF file, 1.5 MB. Copyright © 2019 Piepenbrink et al. 2019 Piepenbrink et al. This content is distributed under the terms of the Creative Commons Attribution 4.0 International license.

To define the ability of the IBV-specific hMAbs to recognize native NA, Madin-Darby canine kidney (MDCK) cells were infected with IAV (A/California/04/09 H1N1) or IBV viruses and hMAb binding evaluated by immunofluorescence assay (IFA). All IBV hMAbs, with the exception of 1122C6, recognized all Victoria and Yamagata lineage IBV tested. Additionally, 1092D4, 1092E10, 1086C12, and 1122C7 also recognized the common ancestor B/Lee/40 virus strain (Fig. 2C). hMAb 1122C6 exhibited the most limited breadth, with substantial binding only to IBV B/Ohio/01/2005 (Victoria lineage)-infected cells consistent with its stronger binding to NA B/Hong Kong/330/2001 by enzyme-linked immunosorbent assay (ELISA) (Fig. 2A). These results indicate that IIV-induced plasmablasts include high-affinity broadly reactive IBV NA-specific hMAbs.

### Molecular characteristics of IBV NA-specific human monoclonal antibodies.

The two hMAbs cloned from subject 134 (1086C12 and 1086F8) may be clonally related as they share the same variable heavy (VH3-30) and lambda light (Vλ1-47) chain gene usage and CDR3 lengths and show 77% HCDR3 and 86% LCDR3 homology (Table 1). The VH chain of hMAb 1086C12 showed higher levels of mutation from the germline VH3-30 than 1086F8, but the Vλ of 1086F8 showed greater mutation from the Vλ1-47 germline than 1086C12. Likewise, hMAbs 1092D4 and 1122C7 are clonally related, sharing the same variable heavy (VH3-23) and light (Vλ6-57) chain gene usage, with similar degrees of mutation from the germline. These two hMAbs also have identical examples of λCDR3 and of HCDR3, differing only in the terminal residue (1092D4 = D, 1122C7 = E). The NA-specific 1092E10 hMAb has the distinction of having the longest (22 amino acids) HCDR3 among the hMAbs. All of the IBV NA-specific hMAbs exhibited modest mutation from germline (VH, 8% to 15% amino acids [aa]; VL, 6% to 11% aa), suggesting that affinity maturation had occurred. Examining the native heavy chain constant region sequence that was expressed by the plasmablast from which the hMAbs were cloned, with the exception of 1122C6, which was IgA1, all were IgG_1_.

**TABLE 1:** Molecular characteristics of IBV NA-specific hMAbs^*a*^

Subject	hMAb	Native isotype	Heavy chain			Light chain		
			Gene usage	Mutation (% NT/% AA)	HCDR3 AA seq	Gene usage	Mutation (% NT/% AA)	LCDR3 AA seq
134	1086C12	IgG1	VH3-30 DH3-22 JH5	9/15	ARDAGCDSVGYYPGRL	Vλ1-47 Jλ2	4/6	AAWDDSLSGHLV
134	1086F8	IgG1	VH3-30 DH3-22 JH4	7/10	ARDAGYDSRGYLPGPY	Vλ1-47 Jλ2	7/10	AAWDDSLSGHVM
105	1092D4	IgG1	VH3-23 DH3-3 JH4	8/9	AKDNQDLDLWSGSYKGTFDD	Vλ6-57 Jλ3	4/7	QSYDSSRYWV
105	1092C7	IgG1	VH3-23 DH3-3 JH4	8/8	AKDKQDLDLWSGSYKGTFDE	Vλ6-57 Jλ3	4/6	QSYDSSRYWV
105	1092E10	IgG1	VH3-15 DH3-16 JH6	6/9	SAAPFTESNGYKSWDYLYGMDV	Vλ3-1 Jλ1	5/11	QAWDSNSYV
105	1092C6	IgA1	VH4-59 DH1-26 JH5	5/10	ARYRVSGNYYDTPWFDP	Vλ3-25 Jλ2	5/9	QSAASSYGYVV

a AA, amino acids; NT, nucleotides.

### Broad and potent *in vitro* antiviral activity of IBV NA-specific hMAbs.

**FIG 3:**
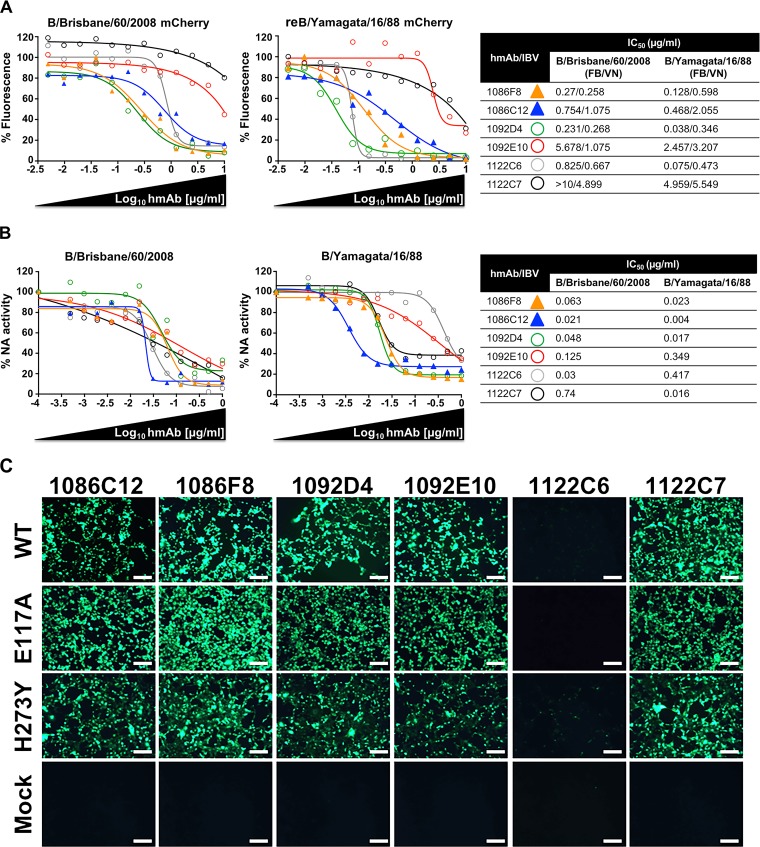
Ability of IBV NA-specific hMAbs to inhibit viral infection and NA activity. (A) Fluorescence-based microneutralization assay. MDCK cells were infected with the indicated mCherry-expressing virus (B/Brisbane/60/2008 or reB/Yamagata/16/1988) and then incubated with 2-fold serial dilutions (starting concentration, 10 µg/ml) of the IBV NA-specific hMAbs. Virus neutralization was evaluated and quantified using a fluorescence microplate reader, and the percentage of infectivity was calculated using sigmoidal dose response curves. Mock-infected cells and viruses in the absence of hMAb were used as internal controls. Percentages of inhibition were normalized to infection in the absence of hMAb. Data show means of the results determined in triplicate. IC_50_ data corresponding to the IBV NA hMAbs were determined using a fluorescence-based assay (FA) or a traditional viral neutralization assay (VN) and mCherry-expressing or WT viruses, respectively. (B) IBV NA-specific hMAbs inhibit NA enzymatic activity. B/Brisbane/60/2008 or B/Yamagata/16/1988 WT viruses were preincubated with 2-fold serial dilutions of the IBV NA-specific hMAbs, and NA activity was determined at 18 h postincubation on fetuin-coated plates. Data represent mean percentages of virus-alone NA activity from duplicate wells. The percentage of activity and the IC_50_ were calculated using sigmoidal dose response curves. (C) IBV NA hMAbs recognize IBV oseltamivir resistance mutations. MDCK cells were infected (MOI of 0.1) with the indicated WT and NA (E117A and H273Y) viruses, and hMAb binding (1 μg/ml) was evaluated by IFA. Bar, 100 µm.

We next evaluated the ability of the 6 identified IBV NA hMAbs to inhibit viral infection by using our recently described fluorescence-based microneutralization assay (43–46). For that, MDCK cells were infected with mCherry-expressing influenza B/Brisbane/60/2008 virus, which is a representative member of Victoria lineage, and then incubated with 2-fold serial dilutions of the IBV NA hMAbs (starting concentration, 10 µg/ml) followed by quantification of the levels of inhibition (Fig. 3A). Notably, hMAbs 1086C12, 1086F8, 1092D4, 1092E10, and 1122C6 displayed dose-dependent inhibition activity against virus infection, although with different levels of efficacy, with 1092E10 being the least potent hMAb. However, hMAb 1122C7 did not show significant inhibition at the concentrations tested in the assay (Fig. 3A).

In order to determine if the inhibitory activity of the IBV NA hMAbs was specific for IBV of the Victoria lineage or if they also have the ability to inhibit IBV of the Yamagata lineage, we used a plasmid-based reverse genetic system (43, 47) to generate a recombinant mCherry-expressing virus containing the six internal genes (PB2, PB1, PA, NP, M and NS-mCherry) from B/Brisbane/60/2008 virus and the HA and NA from influenza B/Yamagata/16/1988 virus, a representative member of the Yamagata lineage. Then, the ability of the six IBV NA hMAbs to inhibit infection was evaluated using our fluorescence-based microneutralization assay as described above. Notably, as we observed previously with mCherry-expressing B/Brisbane/60/2008 virus, the most effective neutralizing hMAbs were 1086C12, 1086F8, 1092D4, and 1122C6. Moreover, hMAbs 1092E10 and 1122C7 showed inhibition only at the higher concentrations (Fig. 3A). The 50% inhibitory concentration (IC_50_) values, determined using a classical sigmoidal dose response curve, showed that the levels of inhibition of the two mCherry-expressing virus strains were similar (Fig. 3A). We then used a conventional virus neutralization (VN) assay to further assess inhibition of influenza B/Brisbane/60/2008 and B/Yamagata/16/1988 wild-type (WT) viruses by the 6 IBV NA-specific hMAbs. The results showed comparable levels of inhibition for the WT and mCherry viruses (Fig. 3A). Moreover, the VN data correlated with the data previously observed in the fluorescence approach as shown by the percentage of inhibition calculated using sigmoidal dose responses (Fig. 3A). hMAb 1092D4 exhibited the greatest potency across all infectivity assays (IC_50_, <0.5 µg/ml) followed by 1086C12 and 1122C6 (IC_50_, <1.0 µg/ml) and 1086F8 (IC_50_, <5.0 µg/ml), with 1092E10 and 1122C7 having the weakest activity.

The main function for viral NA is cleavage of the sialic acid residues on the cell surface, permitting the release of mature virions that then infect new cells. Therefore, to better understand the inhibitory mechanism for the IBV NA hMAbs, the sialic acid cleavage was evaluated by using the enzyme-linked lectin assay (ELLA) with influenza B/Brisbane/60/2008 and B/Yamagata/16/1988 WT viruses as antigens (Fig. 3B). Interestingly, all the hMAbs efficiently inhibited the activity of the viral NA in both viral strains (Fig. 3B). Moreover, we evaluated the ability of the IBV NA hMAbs to bind the NA from two recombinant B/Brisbane/60/2008 virus strains containing amino acid substitution E117A or H273Y (E119A or H274Y [N2 numbering]), which have been previously described to be resistant to oseltamivir (6, 48). For that, MDCK cells infected with WT or mutant viruses were probed with the panel of hMAbs by immunofluorescence assay. All IBV hMAbs recognized the infected cells similarly, independently of the virus used (Fig. 3C). Taken together, these findings suggest that IBV NA hMAbs inhibit virus spread by inhibiting NA enzymatic activity and thus blocking the release of progeny virions from the infected cells, with 1092D4, 1086C12, 1086F8, and 1122C6 being more effective *in vitro* than 1092E10 and 1122C7.

### Efficacy of IBV NA hMAbs in mice.

**FIG 4:**
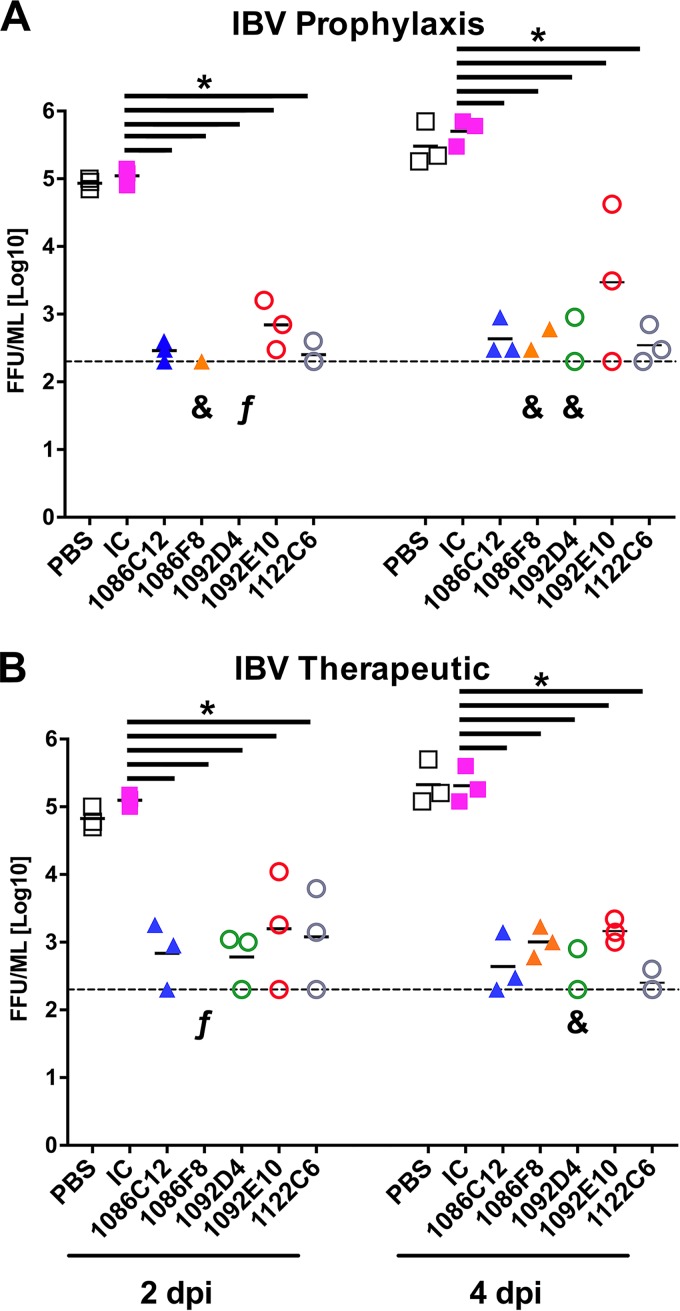
*In vivo* prophylactic and therapeutic activity of the IBV hMAbs. (A) Prophylactic activity. Female C57BL/6 mice (*n* = 3 per group/time point) were administered the indicated IBV NA hMAbs at 20 mg/kg i.p. or the irrelevant isotype control (IC) 1069 D6 hMAb at 20 mg/kg i.p. At 6 h after dosing, mice were inoculated i.n. with 1 × 10^6^ FFU B/Brisbane/60/2008. (B) Therapeutic activity. Female C57BL/6 mice (*n* = 3 per group) were inoculated i.n. with 1 × 10^6^ FFU B/Brisbane/60/2008 and 24 h later were administered the indicated IBV NA hMAb or the irrelevant isotype control (IC) 1069 D6 hMAb at 20 mg/kg i.p. Viral replication (A and B) was determined by measuring viral titers in the lungs of the infected mice at 2 and 4 days p.i. (dpi). Each symbol represents an individual mouse. The “&” symbol indicates that virus was detected in only one or two mice per group. “f” indicates that virus was not detected in any mouse in the group. *, *P* < 0.05 (using one-way ANOVA with multiple-outcome correction).

Given the broad and robust *in vitro* neutralization activity of the IBV NA-specific hMAbs, we next investigated the *in vivo* protective breadth of the IBV NA hMAb panel, using a mouse model of infection (Fig. 4). First, to evaluate the prophylactic efficacy of the IBV NA hMAbs, groups of mice received IBV NA hMAbs 1086F8, 1086C12, 1092D4, 1092E10, and 1122C6; an IgG isotype control hMAb; or phosphate-buffered saline (PBS) only. hMAbs were administered intraperitoneally (i.p.) at 20 mg/kg of body weight, a dose that was selected based on previous studies evaluating other anti-NA MAbs (42, 49) and on the *in vitro* activity that we observed. The hMAbs were given 6 h before intranasal (i.n.) challenge with 10^6^ focus-forming units (FFU) of influenza B/Brisbane/60/2008 WT virus (Fig. 4A). hMAb 1122C7 was not included in these studies since the *in vitro* infectivity assays showed a low level of efficacy against B/Brisbane/60/2008 virus (Fig. 3A). Viral titers in the lungs of the infected mice were determined on days 2 (*n* = 3) and 4 (*n* = 3) postinfection (p.i.) and used as a measure of viral inhibition (Fig. 4A). Mice treated with the IgG isotype control hMAb or with PBS showed high and similar viral titers of ∼10^5^ to 5 × 10^5^ FFU/ml in the lungs at days 2 and 4 p.i. Notably, mice that received the IBV NA hMAbs showed significantly (*P* < 0.05) lower viral titers or no detectable virus in the lungs of infected animals at those times (Fig. 4A). IBV NA hMAbs 1092D4 and 1086F8 were the most effective, with no virus detected in 1092D4-treated mice and virus detected in only one of three 1086F8-treated mice at day 2 p.i. At day 4 p.i., viral replication was lowest in the 1092D4-treated and 1086F8-treated mice, with the level being below the limit of detection in one mouse from each group. Both 1086C12 and 1122C6 suppressed viral replication to 200 to 900 FFU/ml, levels just above detection. The viral load in lungs of mice treated with 1092E10 was on average slightly higher than in animals treated with the other IBV NA hMAbs with higher variability. Those data correlated with the data from our *in vitro* inhibition assays (Fig. 3), where 1092E10 was the less potent hMAb, though still it was efficient with respect to inhibition of influenza B/Brisbane/60/2008 virus infection.

The therapeutic efficacy of the IBV NA hMAbs was also assessed (Fig. 4B). To that end, groups of mice infected with 10^6^ FFU of influenza B/Brisbane/60/2008 WT virus were treated with 20 mg/kg of IBV NA hMAbs at 24 h p.i. In addition, control groups of animals treated with the IgG isotype control hMAb or with PBS were included (Fig. 4B). To assess if the treatment with the IBV NA hMAbs could reduce lung viral loads, viral replication in lungs from infected mice was measured on days 2 (*n* = 3) and 4 (*n* = 3) p.i. Compared to the control treated groups, and correlating with the prophylactic analysis, treatment of mice with IBV NA hMAbs significantly (*P* < 0.05) reduced the level of virus replication in the lungs of the infected mice (Fig. 4B). Taken together, these data indicate that the identified NA hMAbs have potent prophylactic and therapeutic activity against IBV infection *in vivo* and that they can significantly reduce virus dissemination in lungs.

### Clonal persistence of IBV NA-specific hMAbs in bone marrow plasma cells.

**FIG 5:**
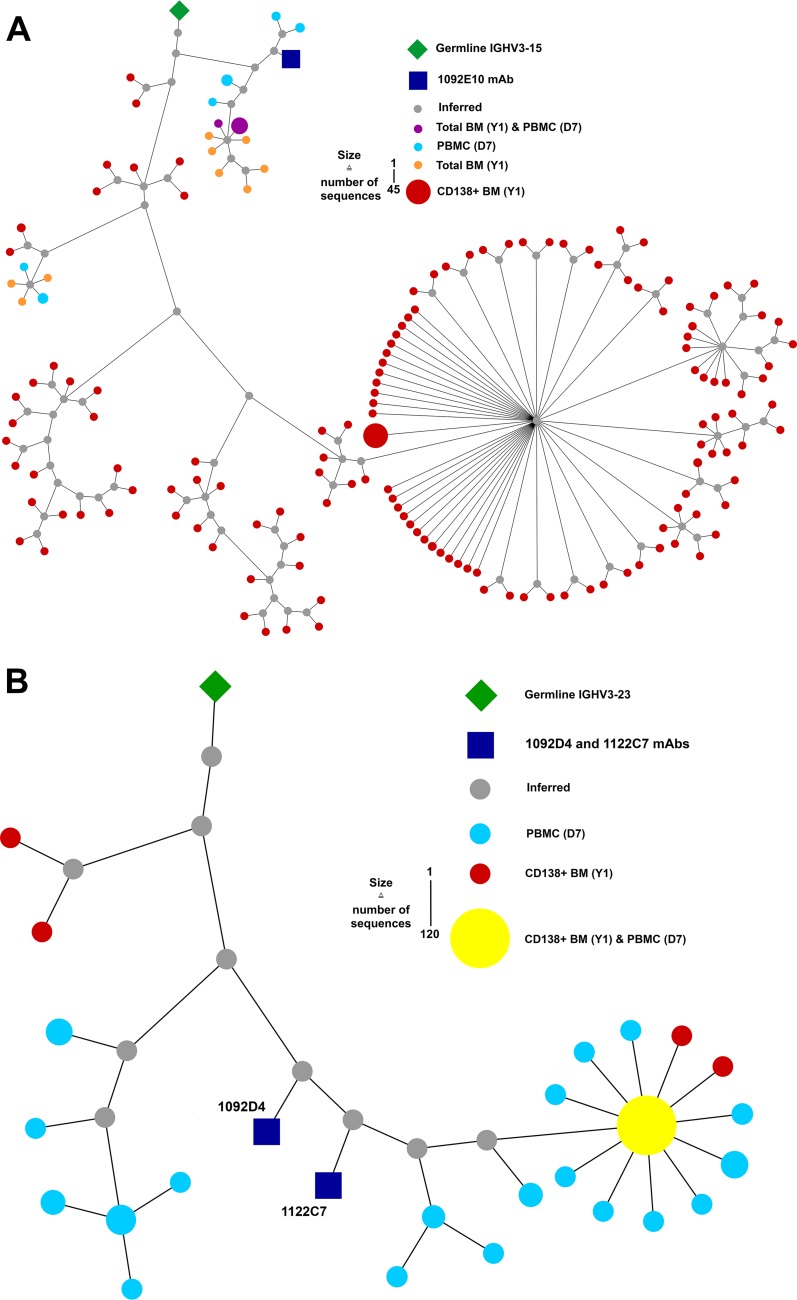
Clonal persistence of IBV NA hMAbs in bone marrow. Phylogenic analysis of 1092E10 (A) and 1092D4 and 1122C7 (B) IBV NA hMAb lineages was based on amino acid sequence. Lineage members are defined as exhibiting the same heavy chain V and J gene usage and HCDR3 lengths and ≥85% HCDR3 similarity. The germline sequence is represented by green diamonds, the MAb sequence is represented by blue squares, sequences obtained by MiSeq-based deep sequencing of bulk total peripheral blood B cells (D7) are represented by teal circles, bulk total bone marrow (BM) B cells (Y1) are represented by orange circles, CD138^+^ bone marrow plasma cells (Y1) are represented by red circles, sequences present in both bulk peripheral blood and bone marrow B cells are represented as purple circles, and inferred intermediate sequences are represented by gray circles. Sizes of symbols are proportional to the number of identical sequences obtained from an individual lineage member (*n* = 1 to 120), with the exception of the germline and hMAb sequences.

Long-lived bone marrow plasma cells are presumed to be the primary source of sustained circulating Abs. To determine if the IBV NA-specific hMAbs isolated from D7 plasmablasts persisted in long-lived bone marrow plasma cells, bone marrow was obtained from subject 105 1 year later, a time at which IBV NA-specific plasma IgG was still detectable (Fig. S2). Bone marrow CD138^+^ plasma cells, total bone marrow B cells, and D7 total peripheral blood B cells were subjected to VH3-targeted deep sequencing of the immunoglobulin repertoire. Members of the 1092E10 clonal lineage were identified among CD138^+^ plasma cells and total bone marrow B cells, with many of the CD138^+^ plasma cells exhibiting additional somatic hypermutation beyond that seen with the 1092E10 hMAb (Fig. 5A). Similarly, members of the 1092D4/1122C7 clonal lineage were found within the CD138^+^ plasma cells, including those with VH sequences identical to those also found in D7 total peripheral blood B cells (Fig. 5B). These results indicate that IBV NA-specific B cell lineages with protective potential persist within the CD138^+^ long-lived bone marrow plasma cell repertoire following IIV immunization.

10.1128/mBio.00066-19.2 FIG S2 Longitudinal IBV HA- and NA-specific plasma IgG. Peripheral blood was collected from subject 105 at baseline and at various time points after immunization with IIV. Plasma was serially diluted at 1:100, 1:500, 1:2,500, and 1:12,500 and tested in triplicate for IgG specific for NA and HA proteins by ELISA, and area under the curve (AUC) data are presented. Download FIG S2, TIF file, 0.4 MB. Copyright © 2019 Piepenbrink et al. 2019 Piepenbrink et al. This content is distributed under the terms of the Creative Commons Attribution 4.0 International license.

## DISCUSSION

Although currently licensed influenza vaccines and antivirals significantly mitigate the morbidity and mortality associated with influenza virus infections, they are suboptimal; consequently, substantial public health vulnerabilities exist with respect to prevention and treatment of influenza. Improving the breadth of activity of both vaccines and antivirals to confer protection from emerging seasonal isolates and from those with pandemic potential is a fundamental area of emphasis. In this study, we demonstrated that IIV induces IBV NA-specific B cells that encode the hMAbs which persist among long-lived bone marrow plasma cells and have broad and potent antiviral activity, including *in vivo* prophylactic and therapeutic activity.

Each of the hMAbs tested bound recombinant and native IBV NA and potently (IC_50_, <0.5 μg/ml) inhibited the NA enzymatic activity of IBV from both the Yamagata and Victoria lineages and, with the exception of 1122C7, also potently (IC_50_, <5 μg/ml) inhibited the replication of IBV from both lineages. This substantial antiviral breadth spanned more than 2 decades of isolates for all the hMAbs and greater than 7 decades for the 1086C12, 1092D4, 1092E01, and 1122C7 hMAbs, which also recognized the ancestral B/Lee/1940 virus isolate, suggesting that sufficiently conserved epitopes are present across a wide range of IBV isolates. Although the epitopes recognized by these hMAbs were not defined, the hMAbs’ inhibition of NA enzymatic activity and yet their ability to recognize IBV with mutations in NA that confer oseltamivir resistance suggest that the hMAbs’ binding is not dependent on E117 or H273 but on other epitopes within the NA active site or within sufficient proximity to the active site to sterically hinder the access of substrate to the active site. Further studies to determine the specific epitopes recognized by the panel of IBV NA-specific hMAbs described here, particularly studies designed to solve directly the crystal structure of the hMAb/NA complexes, are justified and are likely to provide insight into the conserved B cell epitopes of IBV NA to target for universal influenza vaccine development. Recently, Wohlbold et al. (31) described IBV NA-specific MAbs that were isolated from mice immunized with IBV from both Yamagata and Victoria lineages and that had broad activity against IBV and recognized highly conserved epitopes that were outside the active site but in close proximity to it. Similarly, the results suggest that broadly protective IBV NA-specific MAbs can be induced. Follow-up studies to compare the epitopes and activity of the human MAbs that we describe here with those induced in mice would be valuable.

All of the IBV NA-specific hMAbs tested demonstrated prophylactic and therapeutic activity against IBV infection in mice, consistent with their *in vitro* antiviral and NA inhibitory properties. Although a truly stratifying correlation between *in vitro* activity and *in vivo* activity was not resolved, 1086F8 and 1092D4, which exhibited the greatest *in vivo* activity against B/Brisbane/60/2008 virus, including suppressing the virus to below detectable levels in several mice, were the most potent hMAbs in inhibiting B/Brisbane/60/2008 virus replication *in vitro* (IC_50_, <0.5 μg/ml) but were not superior to the other hMAbs in their ELLA activity. Several animal studies demonstrated previously that Fc-mediated effector functions such as ADCC substantially contribute to the *in vivo* antiviral activity of NA-specific antibodies (27–30). Our study did not attempt to define the relative scontribution of Fc-mediated antibody functions; however, our resulting panel of NA-specific hMAbs is likely to serve future utility in defining mechanisms of action of NA Ab-mediated protection.

A significant increase in the levels of IBV NA-specific peripheral blood plasmablasts was evident at D7 following IIV; the increase may have been the consequence of a recall response of preexisting IBV NA-specific memory B cells or of engagement of naive B cells by the IIV. The evidence of moderate somatic hypermutation (VH, 8% to 15%; VL, 6% to 10%) and the timing suggest that it may largely represent a recall response. CD138^+^ long-lived bone marrow plasma cells are presumed to be the major source of serum antibody, and their efficient induction in response to vaccination is likely a critical determinant of sustained protective immunity from pathogens. The clonal persistence of both the 1092D4/1122C7 and the 1092E10 lineages in the bone marrow suggests that the peripheral blood plasmablasts either directly give rise to long-lived CD138^+^ plasma cells or that they share a post-germinal center reaction cellular origin, such as in memory or activated B cells. Additionally, the persistence of both these lineages demonstrates that IBV NA-specific hMAbs with broad antiviral potential are contained within the bone marrow CD138^+^ plasma cell compartment and that their development is not a rare event. However, the precise induction dynamics of such NA-specific long-lived bone marrow plasma cells remains to be defined, as from this single-subject, single time point, it cannot be determined if these plasma cells were present prior to IIV and, if so, whether they developed as a result of immunization or prior infection. Previous analysis of serum IBV NA-specific antibodies by Rajendran et al. (50) demonstrated relatively high titers against IBV NA in adults and the elderly, including against a future isolate they had not yet encountered, similarly suggesting that induction of antibodies against conserved epitopes in IBV NA is not a rare event.

NA is a component of most Food and Drug Administration (FDA)-approved IIV formulations, primarily as a bystander of the manufacturing process that relies on egg-based production of whole virus, with dosing based on HA content and with NA content and quality largely variable and/or undefined (40, 51). Our results demonstrate the ability of IIV to induce IBV NA-specific B cells with broad and potent antiviral activity; however, the consistency at which this occurs and whether these NA-specific B cells and antibodies are of sufficient abundance and are localized to confer protection in humans are unresolved. In recent years, new IIV variations, including intradermal, high-dose, and MF59 adjuvanted forms, have become available that all contain NA and yet might induce distinct NA-specific humoral profiles. It should be noted that a recently FDA-approved IIV, Flublok, which is baculovirus-produced recombinant HA, does not contain NA and may offer true insight into the value or inconsequentiality of NA Abs as its clinical efficacy data accumulate.

Broadly antiviral hMAbs represent an excellent option for effective immunotherapeutics to prevent and treat influenza virus infection for which vaccine-induced immunity has not yet been achieved (representing lack of a vaccine [e.g., pandemic], a suboptimal vaccine, and/or an unvaccinated population) or where existing antiviral drugs are of limited efficacy. A few HA-specific hMAbs have been isolated that have antiviral activity against diverse influenza strains and are in clinical trials for the treatment of hospitalized patients and noncomplicated infections (52–54), highlighting the clinical feasibility and potential of influenza-specific hMAbs. NA-specific hMAbs with broad antiviral activity against IAV have been previously isolated from subjects with acute infection (37); to our knowledge, however, these represent the first IBV NA-specific hMAbs with broad antiviral activity to be described. The hMAbs described here, in particular, 1086F8 and 1092D4, which suppressed virus to below detection, could have clinical potential and warrant further evaluation.

## MATERIALS AND METHODS

### Subjects.

Peripheral blood was obtained from 17 healthy adult subjects prior to, 7 days, and 1 month after receiving the 2014 to 2015 seasonal quadrivalent IIV [Sanofi Pasteur Fluzone; A/California/07/2009 X-179A (H1N1) pdm09, A/Texas/50/2012 X-223A (H3N2), B/Massachusetts/2/2012, and B/Brisbane/60/2008 viruses] as the standard of care at the University of Rochester Medical Center. A 50-ml volume of bone marrow aspirate was obtained from the posterior iliac crest. The subjects provided signed written informed consent. All procedures and methods were approved by the Research Subjects Review Board at the University of Rochester Medical Center, and all experiments were performed in accordance with relevant guidelines and regulations. Peripheral blood mononuclear cells (PBMC) and plasma were isolated using cell preparation tubes (CPT) (Becton, Dickinson, Franklin Lakes, NJ, USA).

### Cells and viruses.

Madin-Darby canine kidney (MDCK; ATCC CCL-34) and human embryonic kidney (HEK293T; ATCC CRL-11268) cells were grown in Dulbecco’s modified Eagle’s medium (DMEM; Mediatech, Inc.) that had been enriched with 5% fetal bovine serum (FBS) and 1% PSG (penicillin, 100 units/ml; streptomycin, 100 µg/ml; l-glutamine, 2 mM) at 37°C with 5% CO_2_ (55).

Recombinant influenza virus A/California/4_NYICE_E3/2009 (pH1N1), B/Yamagata/16/1988, and B/Brisbane/60/2008 WT or mCherry-expressing viruses have been previously described (43, 56, 57). A reassortant IBV containing the six internal genes (PB2, PB1, PA, NP, M, and NS-mCherry) from B/Brisbane/60/2008 virus and the HA and NA from B/Yamagata/16/1988 (reB/Yamagata/16/1988 mCherry) virus was generated using plasmid-based reverse genetics techniques (43, 47). Two recombinant B/Brisbane/60/2008 virus strains containing amino acid substitution E117A or H273Y (E119A or H274Y [N2 numbering]) (6, 48) in the NA were generated using plasmid-based reverse genetics techniques (43, 47). IBV B/Malaysia/2506/2004 (NR-9723), B/Ohio/01/2005 (NR-41801), B/Nevada/03/2011 (NR-44023), B/Sydney/507/2006 (NR-36526), B/Texas/06/2011 (NR-44024), and B/Lee/1940 (NR-3178) were obtained from BEI Resources, and the IBV B/Wisconsin/01/2010 (FR-806) was obtained from International Reagent Resources (IRR). Viral titrations were performed and stocks were produced in MDCK cells at 33°C. For infections, virus stocks were diluted in phosphate-buffered saline (PBS)–0.3% bovine albumin (BA)–1% penicillin-streptomycin (PS) (PBS/BA/PS). After viral infections were performed, cells were maintained in postinfection (p.i.) medium containing DMEM, 0.3% BA, 1% PSG, and 1 μg/ml tosylsulfonyl phenylalanyl chloromethyl ketone (TPCK)-treated trypsin (Sigma) (43, 47). Viral titers were determined by an immunofocus assay using MDCK cells and an anti-HA goat polyclonal antibody (BEI Resources; NR-3165), as previously described (43, 47), and the titers are presented as levels of fluorescence-forming units (FFU) per milliliter.

### Generation and screening of human monoclonal antibodies.

Fresh PBMC collected 7 days after immunization were stained for flow cytometry as previously described (46). Plasmablasts (CD19^+^ IgD^−^ CD38^+^ CD27^++^) were subjected to direct single-cell sorting performed with a FACSAria cell sorter (BD Biosciences) and were placed into 96-well PCR plates (Bio-Rad, Hercules, CA) containing 4 μl of a mixture of 0.5× PBS, 10 mM dithiothreitol (DTT) (Invitrogen), and 8 U RiboLock (Thermo Fisher) RNase inhibitor per well. Plates were sealed with AlumaSeal 96 sealing foil (Excel Scientific, Inc.) and immediately frozen at −80°C until use for reverse transcription-PCR (RT-PCR).cDNA was synthesized, and nested PCR was performed for IgH, Igλ, and Igκ V gene transcripts, followed by linear Ig cassette generation as previously described (58). Human embryonic kidney cells (HEK293T; ATCC CRL-11268) were seeded into 96-well flat-bottom plates at 27,000 cells/well in DMEM–10% HyClone FetalClone II (GE Healthcare Life Sciences, Logan, UT)–1× antibiotic/antimycotic (Gibco, Life Technologies, Grand Island, NY). Cultures achieved 70% to 80% confluence within 48 h of incubation at 37°C with 5% CO_2_. The medium was changed to 100 μl per well of DMEM–2.5% HyClone FetalClone II–1× antibiotic/antimycotic (2.5% FCII). Purified linear cassettes were transfected using jetPRIME transfection reagent (PolyPlus, New York, NY). Approximately 48 h later, an additional 150 μl of 2.5% FCII was added to each well and plates incubated for another 3 days before the medium containing the secreted IgG was harvested. Harvested medium was screened on ELISA plates (Nunc MaxiSorp; Thermo Fisher Scientific, Rochester, NY) coated with 0.5 μg/ml recombinant IBV NA protein (B/Florida/04/2009; BEI Resources, Manassas, VA) and detected with horseradish peroxidase (HRP)-conjugated anti-human IgG (Jackson ImmunoResearch, West Grove, PA). Plates were read at 450 nm using the optical density (OD) readings at 650 nm to subtract background levels. Wells were designated “positive” with OD values greater than 3-fold the OD of the negative control (PBS).

To generate a permanent plasmid containing positive hMAbs, purified PCR products were sequenced at Genewiz Inc. (South Plainfield, NJ) and analyzed by IgBlast (www.ncbi.nlm.nih.gov/igblast) and IMGT/V-QUEST (http://www.imgt.org/IMGT_vquest/vquest) to identify the germline V(D)J gene segments with the highest identity and to determine sequence properties. Expression vector cloning and transfection of human HEK293T cells were performed as previously described (59, 60). IgG was purified from culture supernatant using Magna protein G or A beads (Promega, Madison, WI). 1069 D6 is a human IgG1 MAb and was used as an isotype control.

### Binding characterization (ELISA and avidity).

ELISA plates (Nunc MaxiSorp; Thermo Fisher Scientific, Grand Island, NY) were coated with recombinant NA or HA proteins (BEI Resources, Manassas, VA) at 1 μg/ml or with respiratory syncytial virus (RSV) fusion (F) protein at 0.5 μg/ml, hMAbs or plasma was diluted in PBS, and binding was detected with HRP-conjugated anti-human IgG (Jackson ImmunoResearch, West Grove, PA). Plasma was tested in 5-fold dilutions (1:100 to 1:62,500), and area under the curve (AUC) values were determined. In selected ELISAs, increasing concentrations of urea were added to the ELISA plate and the plates incubated for 15 min at room temperature prior to detection with anti-IgG-HRP to evaluate avidity.

### Virus neutralization and fluorescence-based microneutralization assays.

Virus neutralization assays were performed with WT and mCherry-expressing viruses as previously described (43–46). Briefly, confluent monolayers of MDCK cells (5 × 10^4^ cells/well, 96-well plate format, triplicates) were infected with 200 FFUs of indicated viruses. After 1 h viral adsorption, cells were maintained at 33°C in p.i. medium supplemented with 1 μg/ml TPCK-treated trypsin and 2-fold serial dilutions of the indicated hMAbs (starting concentration, 10 µg/ml). For the fluorescence-based microneutralization assays, at 48 to 72 h p.i., cell monolayers were washed with PBS prior to red fluorescence quantification using a fluorescence plate reader (DTX-880; Becton Dickenson). Fluorescence values of mCherry virus-infected cells in the absence of hMAb were used to calculate 100% viral infection. Cells in the absence of viral infection were used to calculate the fluorescence background. WT virus neutralization was determined by crystal violet staining at 96 to 120 h p.i. Triplicate wells were used to calculate the mean and SD of neutralization, and 50% inhibitory concentrations (IC_50_) were determined with a sigmoidal dose response curve (Graphpad Prism, v7.0).

### Enzyme-linked lectin assay (ELLA).

The ability of IBV NA hMAbs to inhibit the activity of the viral NA was measured using a standard ELLA as previously described (16, 61–63). Briefly, 2-fold serial dilutions of the hMAbs (starting concentration, 1 µg/ml) were preincubated with B/Brisbane/60/2008 or B/Yamagata/16/1988 WT viruses at a predetermined concentration of virus for 2 h at room temperature in Dulbecco’s PBS (DPBS) (Gibco) supplemented with 1% bovine serum albumin (BSA) (diluent buffer). Virus-hMAb dilutions were added to 96-well plates coated with 50 µg/ml of fetuin (Sigma) and incubated for 18 h at 37°C. Then, plates were extensively washed with PBS containing 0.05% Tween 20 and incubated with HRP-coupled peanut lectin agglutinin (Sigma) in diluent buffer for 2 h at room temperature. After washing of the plates with PBS-Tween, the reactions were developed with 3′,5,5′-tetramethylbenzidine (TMB) substrate (BioLegend) for 15 to 20 min at room temperature, quenched with 2 N H_2_SO_4_, and read at 450 nm (Vmax kinetic microplate reader; Molecular Devices). The IC_50_ was determined with a sigmoidal dose response curve (Graphpad Prism, v7.0).

### Immunofluorescence assay (IFA).

Confluent monolayers of MDCK cells (2 × 10^5^ cells/well, 24-well plate format) were mock infected or infected (multiplicity of infection [MOI] of 0.1) with the indicated WT viruses. At 17 h p.i., cells were fixed with 4% paraformaldehyde (PFA) and permeabilized with 0.5% Triton X-100–PBS for 15 min at room temperature. Cells were then incubated for 1 h at 37°C with 1 μg/ml of IBV NA-specific hMAbs or with IAV HA hMAb KPF1 (46) as a control. Then, cells were incubated with fluorescein isothiocyanate (FITC)-conjugated secondary anti-human Ab (Dako) for 1 h at 37°C. Images were captured using a fluorescence microscope (Olympus IX81) and camera (QImaging, Retiga 2000R) with a 10× objective.

### Antibody-secreting cell enzyme-linked immunosorbent spot (ELISpot) assays.

The frequency of influenza antigen-specific antibody-secreting cells (ASCs) was measured by ELISpot assay as previously described (64). Briefly, ELISpot assay plates were coated overnight with either recombinant NA (5 μg/ml B/Florida/04/2009 or B/Hong Kong/330/2001 virus; BEI Resources, Manassas, VA, USA) or Fluzone IIV vaccine (6 μg/ml, Sanofi Pasteur Inc., Swiftwater, PA, USA) and incubated at 37°C for ∼40 h with 500,000 or 100,000 PBMC. Bound antibodies were detected with alkaline phosphatase-conjugated anti-human IgG (Jackson Immunoresearch) (1 μg/ml). Spots in each well were counted using a CTL immunospot reader (Cellular Technologies Ltd., Shaker Heights, OH, USA).

### Prophylactic and therapeutic protective activities of NA-specific hMAbs in mice.

Female C57BL/6 mice (5 to 7 weeks of age) were purchased from the National Cancer Institute (NCI) and maintained in the animal care facility at the University of Rochester under specific-pathogen-free conditions. All animal protocols were approved by the University of Rochester Committee of Animal Resources and complied with the recommendations in the Guide for the Care and Use of Laboratory Animals of the National Research Council (65). For viral infections, mice were anesthetized intraperitoneally (i.p.) with 2,2,2-tribromoethanol (Avertin; 240 mg/kg of body weight) and were then inoculated intranasally (i.n.) with 10^6^ FFU of influenza B/Brisbane/60/2008 WT virus in a final volume of 30 µl. To determine the prophylactic efficacy of the NA hMAbs, at 6 h before infection, mice (*n* = 6) were subjected to i.p. administration of 20 mg/kg of the hMAbs, an irrelevant isotype control 1069 D6 hMAb (46), or PBS. For the study of therapeutic efficacy, at 24 h p.i., groups of mice (*n* = 6) were given i.p injections of 20 mg/kg of the indicated NA hMAbs, the isotype control IgG, or PBS. Viral replication was determined in the lungs of the infected mice at days 2 and 4 p.i. To determine the levels of replication, three mice from each group were euthanized by administration of a lethal dose of avertin and exsanguination and lungs were surgically extracted and homogenized. Virus titers (FFU per milliliter) were determined by immunofocus assay as indicated above (43, 46, 47). Geometric mean titers and data representation were performed using GraphPad Prism (v7.0).

### Deep-sequencing immunoglobulin repertoire analysis.

PBMC were isolated from whole blood collected into CPT as described above. In addition to the samples collected 7 days after immunization, PBMC were also isolated from blood samples collected more than 2 months prior to vaccination, 7 weeks after vaccination, and more than 15 months after vaccination. For the final blood sample, approximately 50 million PBMC were used to enrich for B cells by using biotinylated anti-CD3, anti-CD4, and anti-CD14 antibodies along with antibiotin microbeads (Miltenyi Biotec, Auburn, CA) in a negative selection. Bone marrow aspirate was obtained at more than 12 months following the influenza vaccination, and mononuclear cells were isolated by floating the cells over Ficoll-Paque Plus medium (GE Healthcare BioSciences, Pittsburgh, PA). Approximately 40 million cells were then used with CD138 microbeads (Miltenyi Biotec) to isolate the CD138-positive fraction according to the manufacturer’s protocol. The entire positive fraction was lysed in RLT buffer (Qiagen, Hilden, Germany; catalog no. 79216) and stored at −80°C until RNA isolation could be performed. RNA was isolated from all samples using an RNeasy Minikit (Qiagen), treated with DNase I (Turbo DNA-free kit; Invitrogen, Vilnius, Lithuania), and used to synthesize cDNA with a qScript cDNA synthesis kit (QuantaBio, Beverly, MA). The resulting cDNA was used in subsequent PCR using Platinum *Taq* high-fidelity polymerase (Invitrogen, Carlsbad, CA) as previously described (46). Targeted PCR was performed to try to detect lineage members of the cloned monoclonal antibodies by using forward primers specific for VH3-15 (TAARAGGTGTCCAGTGT) and VH3-23 (AGTTTGGGCTGAGCTGGCTT). Gel-extracted PCR products were submitted to the University of Rochester Genomics Research Center, where Qubit fluorometric quantitation (Thermo Fisher) and Bioanalyzer (Agilent Technologies, Santa Clara, CA) sizing, quantitation, and quality control were performed prior to normalization to 2 nM and flow cell hybridization and cluster generation for a MiSeq system (Illumina, Inc., San Diego, CA). Paired-end reads (300 by 325 bp) were made. Sequence analysis and assembly of lineage trees were performed using an in-house custom analysis pipeline as previously described (46, 66). All sequences were aligned using IMGT.org/HighVquest (67). Lineage trees were generated by identifying the lineage (the cluster of sequences with identical VH, JH, and HCDR3 lengths and ≥85% HCDR3 similarity) containing the corresponding MAb sequence. Sequences within a lineage with single occurrences of particular VDJ nucleotide sequences (singletons) were removed, with the exception of singletons obtained from CD138^+^ bone marrow samples. The resulting sequences were analyzed using Phylip’s protpars tool (version 3.695) (68), turning on settings 1, 4, and 5. The output file was then parsed using in-house custom scripts, collapsing any duplicate inferred sequences into an individual node, and was visualized using Cytoscape (69).

### Statistical analysis.

Significance was determined using GraphPad Prisim, v7.0. Paired *t* tests were applied for evaluation of the results of the serum binding antibody assessment, and the Mann-Whitney test was used for evaluation of the results of the ELISpot assessment. One-way analysis of variance (ANOVA) was used to determine the statistical significance of the *in vivo* viral titers.

### Data availability.

All study data are contained within the paper or supplemental materials.
