# Supplementary figures and images for: Broad and Protective Influenza B Virus Neuraminidase Antibodies in Humans after Vaccination and their Clonal Persistence as Plasma Cells
Source: mBio. 2019 Mar 12;10(2):e00066-19. doi: 10.1128/mBio.00066-19 (PMC6414695; doi:10.1128/mBio.00066-19)

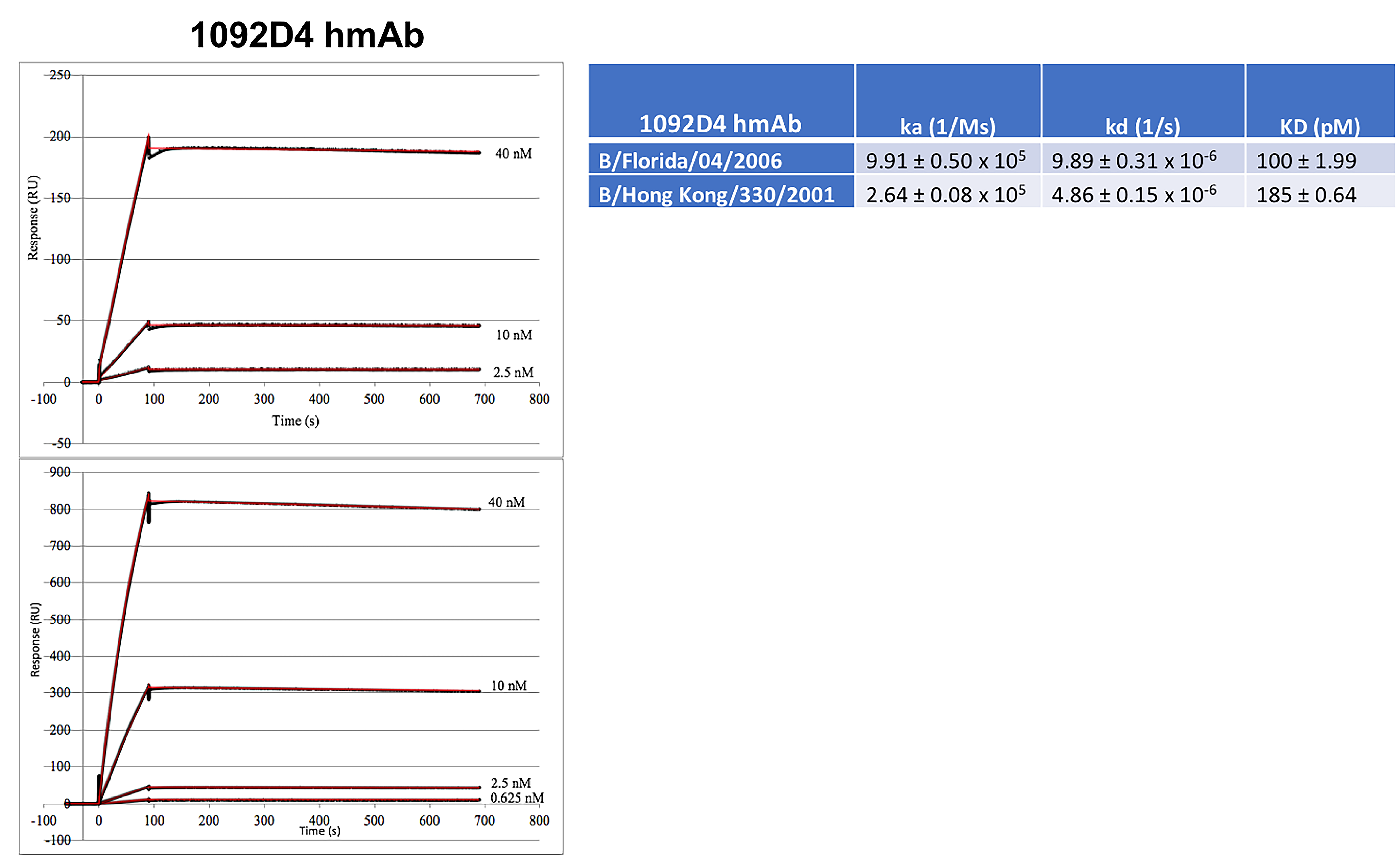

Supplement: FIG S1 [file mBio.00066-19-sf001.tif]

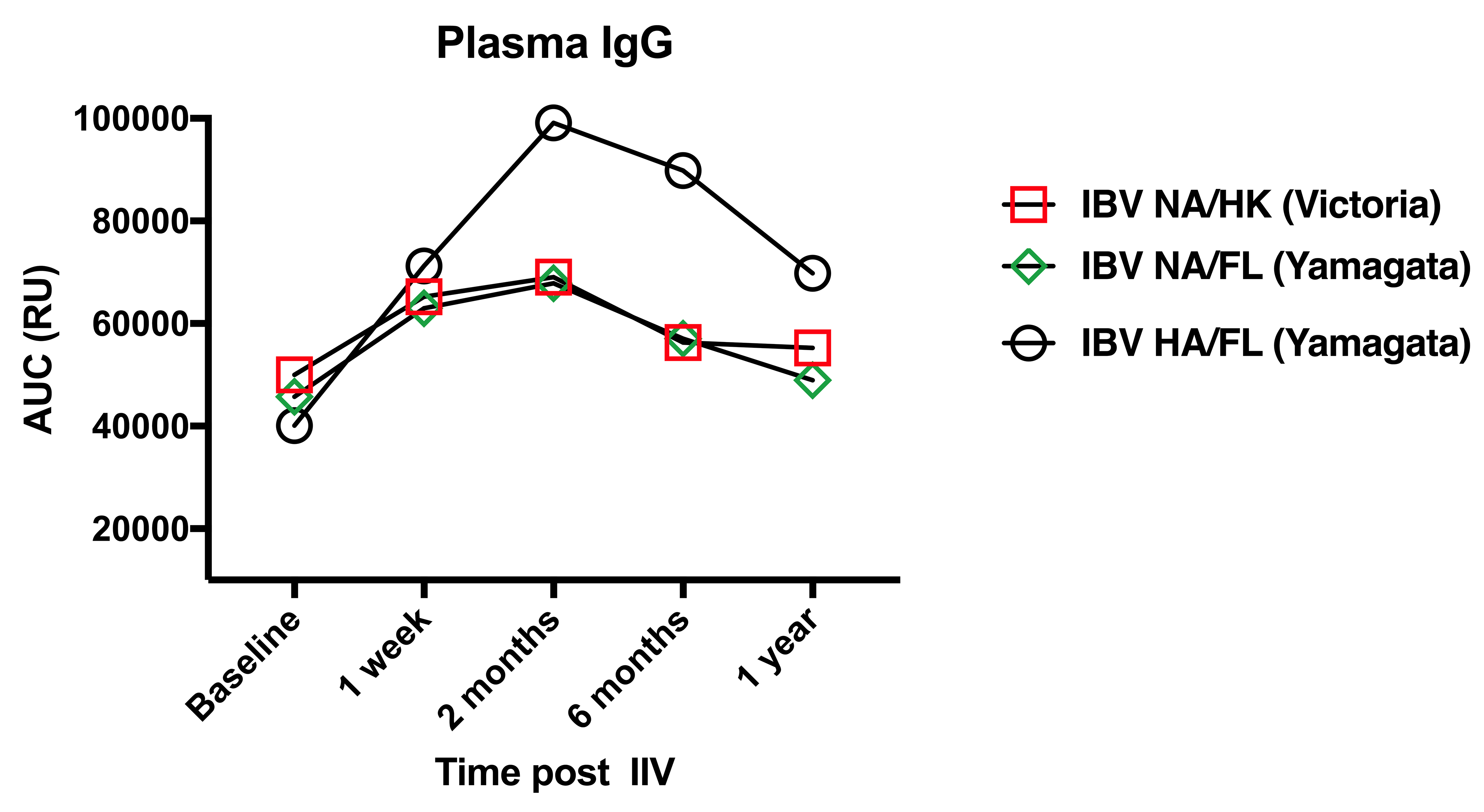

Supplement: FIG S2 [file mBio.00066-19-sf002.tif]
